# An association study of C9orf3, a novel component of the renin-angiotensin system, and hypertension in diabetes

**DOI:** 10.1038/s41598-020-73094-0

**Published:** 2020-09-30

**Authors:** Mai Ichikawa, Tadashi Konoshita, Yasukazu Makino, Jinya Suzuki, Tamotsu Ishizuka, Hiroyuki Nakamura

**Affiliations:** 1 Third Department of Internal Medicine, University of Fukui Faculty of Medical Sciences, 23-3, Matsuokashimoaizuki, Eiheiji, Fukui 910-1193 Japan; 2 Department of Environmental and Preventive Medicine, Kanazawa University Graduate School of Medical Science, Kanazawa, Japan

**Keywords:** Genetics, Cardiology, Endocrinology

## Abstract

The renin-angiotensin system (RAS) is important in the onset and course of cardiovascular, kidney, and metabolic disorders. Previous reports showed that the RAS blockade protects organs and suppress the development of type 2 diabetes mellitus. A novel component of the RAS, namely, chromosome 9 open reading frame 3 (C9orf3), was recently identified, however, its effects are unclear. We evaluated whether the genetic variant of C9orf3 is associated with morbidity of hypertension among subjects with type 2 diabetes. We enrolled 382 subjects with type 2 diabetes, 222 of whom were diagnosed with hypertension. Human leukocyte genomic DNA was isolated and a genetic variant was analyzed for a C/T variant of C9orf3 (rs4385527) via PCR analysis. The relationship between the genotype and hypertension morbidity among subjects with diabetes was examined. The proportion of the respective C9orf3 genetic variants were as follows 247 CC, 119 CT, and 16 TT. The risk of hypertension was determined to be 1.58, with a 95% confidence interval of 1.11–2.27. Moreover, the p value was 0.012 for allelic comparison and for Armitage’s trend test, with the C allele identified as the risk factor. Consequently, hypertension was markedly associated with type 2 diabetes in subjects with the C9orf3 variant, exhibiting a nearly 1.6-fold increased risk. The C variant of a new component of the RAS, C9orf3 (rs4385527) might have a considerable impact on the pathogenesis of hypertension in diabetes.

## Introduction

Excessive activation of the renin-angiotensin system (RAS) plays a pivotal role in the onset and course of cardiovascular^1^, kidney^2^, and metabolic disorders^3^. The RAS also functions in blood pressure regulation and electrolyte metabolism^4^. Moreover, a specific genotype of the RAS has been shown to contribute to tissue expression, plasma renin activity (PRA) in combination with environmental factors^5^ and angiotensin receptor blockers^6^. Previous studies have reported that RAS inhibitors exhibit organ-protective effects^7–9^ while suppressing the onset of type 2 diabetes mellitus^10–12^. In 2014, we reported that the aldosterone synthase (CYP11B2) C-344T variant was markedly associated with type 2 diabetes morbidity^13^. Furthermore, multivariate logistic-regression analysis indicated that age, gender, and CYP11B2 genotype were independently associated with type 2 diabetes; and the risk associated with development of type 2 diabetes when individuals had TT haplotype compared to CC/CT was determined to be 1.40 [95% confidence interval (CI): 1.04–1.90, *p* = 0.029].

Recently, a novel component of the RAS, namely, chromosome 9 open reading frame 3 (C9orf3), which is an aminopeptidase cleaving angiotensin III-generating angiotensin IV, was identified in addition to alanyl aminopeptidase^14,15^. This aminopeptidase was also called aminopeptidase O, an intracellular member of the M1 metalloprotease family of enzymes. This protein catalyzes the hydrolysis of amino acids and N-terminal residues of peptides or protein substrates. It is expressed in the heart, testis, placenta, liver, and pancreas. C9orf3 was suggested to affect the concentrations of angiotensin-related molecules in tissues. Thus, C9orf3 may be involved in the development of specific clinical conditions. Specifically, a marked relationship between the C9orf3 (rs4385527) genotype and polycystic ovarian syndrome (PCOS) has been reported^16^, which is a condition that affects hyperandrogenism and is associated with metabolic disorders. PCOS also raises the risk of type 2 diabetes and cardiovascular diseases^17,18^, with higher rates of morbidity associated with hypertension in women with PCOS compared to their healthy counterparts^19^. In this study, the GG haplotype, or CC haplotype, was markedly higher in the PCOS patients than in controls, revealing that the CC haplotype is a risk factor for PCOS^17,18^.

However, the role of C9orf3 in hypertension has not yet been examined. We, therefore, sought to determine whether specific genetic variant of C9orf3 was associated with the morbidity of hypertension among subjects with type 2 diabetes.

## Methods

### Subjects

Without any specific inclusion criteria we enrolled 382 subjects who presented at our hospitals with type 2 diabetes between November 2000 and November 2013. The study was approved by the ethics committee of Fukui University (n. 13.1/14.2) and written informed consent was obtained prior to enrollment of all participants. The study has been registered with UMIN-CTR (#UMIN000001580). The study was conducted in accordance with the International Ethical Guidelines and Declaration of Helsinki. Diabetes was diagnosed according to the criteria established by the World Health Organization. Patients with fasting plasma glucose ≥ 126 mg/dL, 2-h post-load plasma glucose after a 75 g oral glucose tolerance test ≥ 200 mg/dL, glycosylated hemoglobin A1c ≥ 6.5%; or a random blood glucose ≥ 200 mg/dL in the presence of signs and symptoms are considered to diabetes^20^. Subjects aged less than 20 years and those with type 1 diabetes mellitus, gestational diabetes mellitus, secondary diabetes mellitus, severe organ failure, or acute phase disorders were excluded. At the time of blood sampling, no subjects had been treated with anti-hypertensive or anti-dyslipidemic agents in the previous one week. Subjects with diabetes continued to receive their usual care. Following the Japanese guideline, hypertension is defined as systolic blood pressure (SBP) of ≥ 140 mmHg or diastolic blood pressure (DBP) of ≥ 90 mmHg, measuring at an examination room^21^.

### C9orf3 genotyping

Genotyping was carried out with genomic DNA isolated from human leukocytes using a commercial kit (QIAamp DNA Blood Mini Kit QIAGEN, Hilden, Germany), as previously reported^13^. A genetic variant was analyzed using genomic DNA for a C/T variant of C9orf3 (rs4385527) with the StepOnePlus real-time PCR system and the TaqMan method (Applied Biosystems, Foster City CA, USA). The probe mix (primer) of rs4385527 was TaqMan SNP Genotyping Assay (Assay ID: C___7961143_20). The global minor allele frequency of C9orf3 (rs4385527) was 0.23 (T allele). The Japanese minor allele frequency of C9orf3 (rs4385527) was 0.21 (T allele). The real-time PCR was analyzed by using StepOnePlus software version 2.2.2. The PCR program consisted of Pre-PCR Read (Holding Stage 1) at 50 °C for 2 min, Holding Stage 2 at 95 °C for 10 min, 45 cycles of Cycling Stage (denaturing at 92 °C for 15 s followed by annealing and extension at 60 °C for 1 min), and post-PCR Read (Holding Stage 3) at 60 °C for 30 s.

### Statistical analysis

This statistical analysis was carried out as previously reported^13^. Briefly, the sample size of the study was calculated by setting the difference to be detected between gene groups as at least 10% for hypertension prevalence. By using χ^2^ analysis with a 5% significance level and 80% power, we determined that the study required approximately 400 subjects in total. All statistical analyses were conducted using SPSS Version 22.0 software (SPSS, Inc., St. Louis, MO, USA). The allele frequencies for each genotype were tested by contingency table analysis. A *p* < 0.05 was regarded as statistically significant. Data were presented as numbers/percentages, means ± SD, or medians (interquartile ranges), as appropriate. The differences between 2 paired continuous variables were analyzed by Student’s *t-*test or Wilcoxon signed rank test as appropriate. Dichotomous characteristics were compared by χ^2^ analysis for tests including Hardy–Weinberg equilibrium. Odds ratios for hypertension and 95% CIs were calculated using Armitage’s trend test.

## Results

Overall, 382 subjects were included in this cohort, of which 222 (58.1%) were hypertension cases. Moreover, 227 males (59.4%) and 155 females (40.6%) were enrolled. The mean age of the participants was 61.9 ± 12.0 years, mean Body Mass Index (BMI) was 24.2 ± 4.0, fasting plasma glucose was 155.6 ± 65.6 mg/dL, glycosylated hemoglobin A1c was 7.6 ± 1.5%, estimated glomerular filtration rate was 79.0 ± 26.3 mL/min/1.73 m^2^, and urinary albumin excretion rate was 23.0 (9.0–75.4) mg/g CRE. Mean systolic blood pressure and diastolic blood pressure were 143.8 ± 21.1 mmHg and 82.6 ± 12.4 mmHg, respectively.

We compared the baseline characteristics of subjects who were non-hypertensive to those that were hypertensive (Table 1). Between the two groups, no differences were observed in gender, age, fasting plasma glucose, and glycosylated hemoglobin A1c. However, the BMI and urinary albumin excretion rate in the hypertensive cases were significantly higher than in the controls. Alternatively, the estimated glomerular filtration rate was significantly higher in the controls than in the hypertensive cases.

**Table 1:** Comparison of characteristics between subjects who were non-hypertensives and those who were hypertensives.

Characteristics	Controls	Cases	*p* value
Number	160	222	
Gender (male)—%	62.5	57.2	0.300
Age (≥ 65 years)—%	34.4	43.2	0.081
BMI (≥ 25)—%^a^	27.7	42.1	0.004
FPG—mg/dL^b^	161.8 ± 79.7	151.1 ± 52.8	0.119
Glycosylated hemoglobin—%^b^	7.8 ± 1.7	7.5 ± 1.4	0.035
eGFR—mL/min/1.73 m^2b^	84.1 ± 27.3	75.4 ± 14.9	0.001
U-Alb/Cr ratio—mg/g CRE^c^	13.2 (6.0–39.4)	33.5 (13.1–156.5)	< 0.001

Hypertension is defined as systolic blood pressure (SBP) of ≥ 140 mmHg or diastolic blood pressure (DBP) of ≥ 90 mmHg.

*FPG* fasting plasma glucose, *eGFR* estimated glomerular filtration rate, *U-Alb/Cr ratio* urine albumin-to-creatinine ratio.

^a^Body Mass Index (BMI) is the weight in kilograms divided by square of the height in meters.

^b^Values are means ± SD.

^c^Values shown are medians (interquartile ranges).

Within all of the type 2 diabetic patients, the proportion of the C9orf3 haplotype variants were as follows CC (247), CT (119), and TT (16). Moreover, in hypertensive patients, CC accounted for 154 cases, CT for 62 cases and TT for 6 cases. In non-hypertensive controls, CC accounted for 93 cases, CT for 57 cases, and TT for 10 cases. The distributions were similar to the expected values according to Hardy Weinberg equilibrium. The risk of hypertension was determined to be 1.58, with the 95% confidence interval at 1.11–2.27. The p-value was 0.01 for allelic comparison as well as for the Armitage’s trend test, with the C allele found to be a significant risk factor. Consequently, a significant association with hypertension among subjects with type 2 diabetes was observed for the C9orf3 variant with a 1.6-fold increased risk (Table 2).

**Table 2:** Analysis results.

	C	T
Cases	370	74
Controls	243	77
Odds ratio	1.58	
(95% CI)	(1.11–2.27)	
	C to T	
*p* value	0.01	
Armitage’s trend test *p* value	0.01	

**Figure 1:**
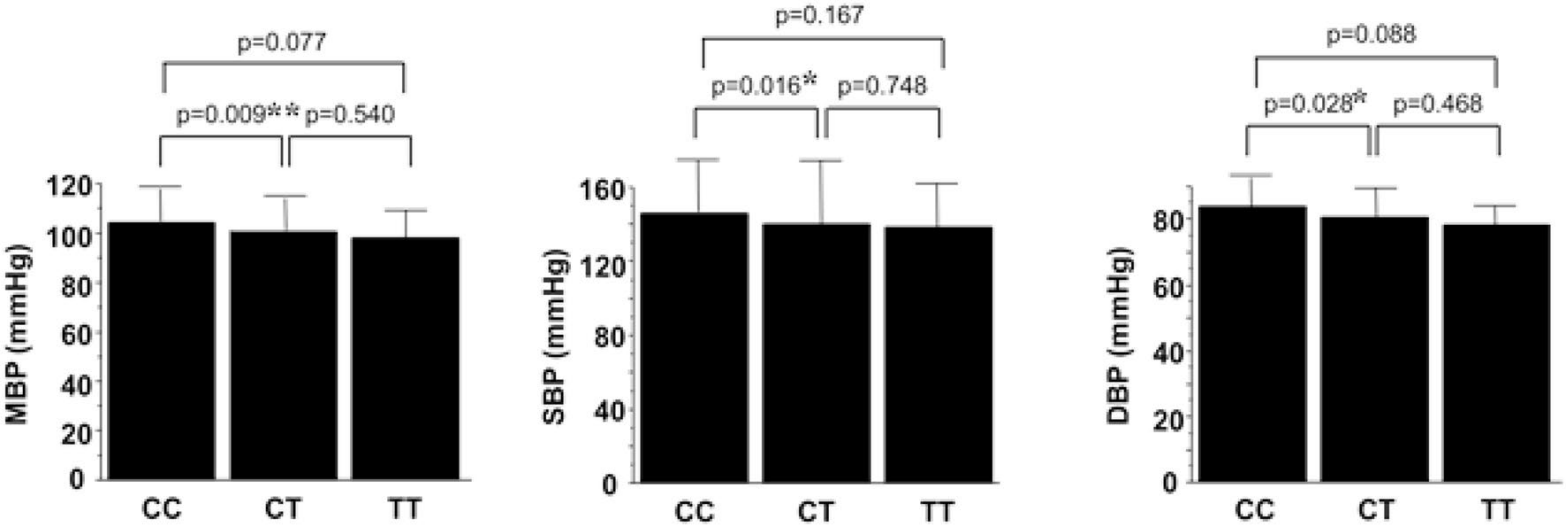
C9orf3 genotypes and corresponding blood pressure readings. Closed columns and bars indicate the means and standard deviations of blood pressure readings. For mean blood pressure (MBP), the values of each genotype were as follows: CC, 104.5 ± 13.5, CT, 100.6 ± 13.5, and TT, 98.4 ± 11.2 mmHg, respectively. For systolic blood pressure (SBP), the values of each genotype were as follows CC, 146.0 ± 20.7, CT, 140.2 ± 21.9, and TT, 138.4 ± 18.2 mmHg, respectively. For diastolic blood pressure (DBP), the values of each genotype were as follows CC, 83.8 ± 12.6, CT, 80.8 ± 12.1, and TT, 78.4 ± 10.4 mmHg, respectively. *p < 0.05 for the comparison with CC; **p < 0.01 for the comparison with CC.

Furthermore, associations were observed between each genotype of C9orf3 and blood pressure readings. Mean blood pressure levels in subjects with each genotype were as follows CC (104.5 mmHg), CT (100.6 mmHg), and TT (98.4 mmHg) with a p value of 0.009 when comparing the blood pressure of patients with CC to those with CT. Systolic blood pressure levels in subjects with each genotype were as follows: CC, 146.0 mmHg; CT, 140.2 mmHg, and TT, 138.4 mmHg (*p* = 0.016, CC to CT), while the diastolic blood pressure levels in subjects with each genotype were, CC, 83.8 mmHg; CT, 80.8 mmHg; and TT, 78.4 mmHg (*p* = 0.028, CC to CT). For all blood pressure values, the CC allele tended to be associated with high blood pressure compared to the CT and TT alleles (Fig. 1).

There were no associations between each C9orf3 genotype and BMI, glycosylated hemoglobin A1c, estimated glomerular filtration rate, or urinary albumin excretion rate. Additionally, there were no significant associations observed between each C9orf3 genotype and the aldosterone concentration and angiotensin II concentration. However, among the RAS components, significant associations were observed between each C9orf3 genotype and PRA. PRA values for both the CT and TT alleles were significantly higher than for the CC allele (Table 3).

**Table 3:** Comparison of characteristics between CC, CT, and TT.

Characteristics	CC	CT	TT
Number	247	119	16
Gender (male)—%	62.3	52.1	68.8
Age (≥ 65 years)—%	40.5	40.3	18.8
BMI (≥ 25)—%^a^	36.4	34.7	43.8
FPG—mg/dL^b^	153.1 ± 54.3	159.8 ± 83.0	162.3 ± 78.6
Glycosylated hemoglobin—%^b^	7.5 ± 1.4	7.7 ± 1.8	8.2 ± 1.7
eGFR—mL/min/1.73 m^2b^	78.8 ± 26.6	72.3 ± 23.4^d^	79.7 ± 36.4
U-Alb/Cr ratio—mg/g CRE^c^	23.6 (9.3–67.5)	18.6 (7.8–119.3)	52.5 (11.3–188.8)
PRA—ng/mL/h^c^	0.60 (0.30–1.10)	0.80 (0.50–1.70)^d^	1.05 (0.90–1.30)^d^
Angiotensin II—pg/mL^c^	9.0 (6.0–14.0)	9.0 (6.0–14.0)	12.0 (8.3–19.5)
PAC—pg/mL^b^	81.6 ± 41.2	84.9 ± 35.2	81.7 ± 30.3

*FPG* fasting plasma glucose, *eGFR* estimated glomerular filtration rate, *U-Alb/Cr ratio* urine albumin-to-creatinine ratio, *PRA* plasma renin activity, *PAC* plasma aldosterone concentration.

^a^Body Mass Index (BMI) is the weight in kilograms divided by square of the height in meters.

^b^Values are means ± SD.

^c^Values shown are medians (interquartile ranges).

^d^*p* < 0.05 for the comparison with CC.

## Discussion

Recently, genetic variants of specific RAS components have been examined to determine their associations with phenotypic characteristics in numerous physiological and pathological conditions, particularly cardiovascular and renal disorders^22–27^. However, the association between C9orf3 and cardiovascular or renal disorders has not been widely studied.

C9orf3 is an aminopeptidase-generating angiotensin IV from angiotensin III and facilitates the conversion of angiotensin II, the primary effector protein of the RAS^28^. In 2011, it was reported that a meta-analysis of genome-wide association studies revealed that genetic variants of glutamyl aminopeptidase were associated with blood pressure variations in East Asians^29^. This enzyme also facilitates the conversion of angiotensin II to angiotensin III. Thus, the C9orf3 gene may also be involved in cardiovascular conditions. The rs4385527 of C9orf3 was previously shown to be associated with PCOS, which is well-known to be strongly related to insulin resistance, obesity, and hypertension^30^. In this study, the rs4385527 genotype of C9orf3 was found to be associated with the morbidity of hypertension among subjects with type 2 diabetes.

**Figure 2:**
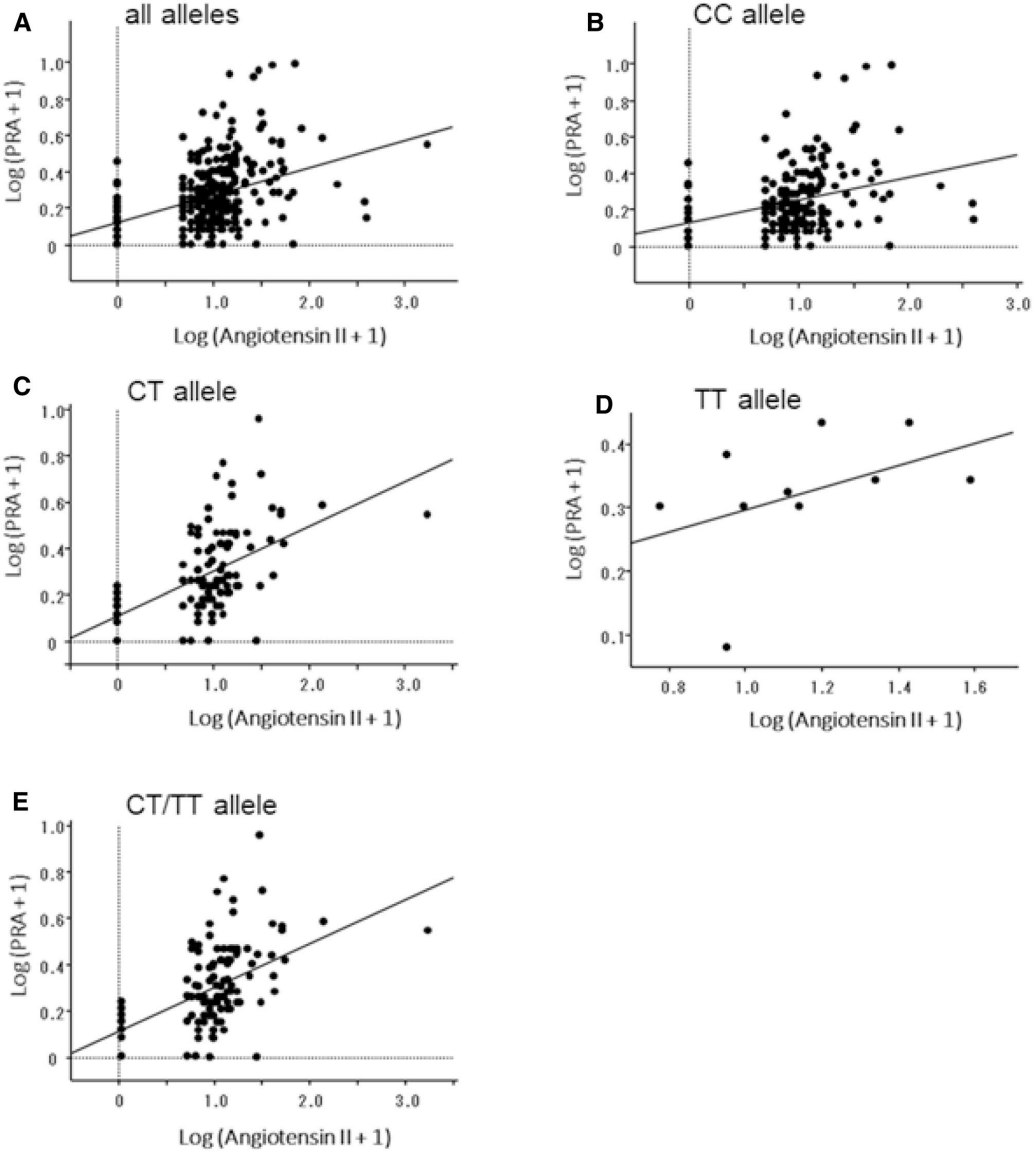
Scatter diagrams of angiotensin II vs plasma renin activity (PRA) in each allele. All values were logarithmically transformed. A: in all alleles. r = 0.374. *p* < 0.0001. B: in CC allele. r = 0.305. *p* < 0.0001. C: in CT allele. r = 0.491. *p* < 0.0001. D: in TT allele. r = 0.438. *p* = 0.1784. E: in CT/TT allele. r = 0.371. *p* < 0.0001.

The rs4385527 is a polymorphism within the intron region, and the C allele may influence the angiotensin II concentration. In our cohort, PRA for both the CT and TT alleles was significantly higher than for the CC allele. Thus, the CC allele was determined to constitute a risk allele; and it is believed that the angiotensin II concentration for the CC allele is higher than that for the CT and TT alleles. This indicates that the decreased PRA for the CC allele is under negative feedback control^31^. According to our evaluation of the relation between angiotensin II and PRA in logarithmic transformed values on scatter diagrams, we found that there is a positive correlation between angiotensin II and PRA. The correlation was weaker in the CC allele (r = 0.305) compared with CT/TT alleles group (r = 0.371) as well as all subjects (r = 0.374), as we expected (Fig. 2). In other words, we presume that the CC allele reduces the generation of angiotensin IV from angiotensin III compared with the CT and TT alleles, and the increased accumulation of angiotensin III inhibits the degradation of angiotensin II. The angiotensin II concentration rises and activates the RAS, thereby causing hypertension. However, no associations were observed between each genotype of C9orf3 and the circulating angiotensin II concentration. This result for PRA may be related to differences in the tissue angiotensin II concentration. To determine the associations between each C9orf3 genotype and circulating angiotensin II concentration, a larger sample size is needed.

Our cohort included only subjects with type 2 diabetes. Although subjects carrying the CC allele without diabetes were prone to have a higher prevalence of hypertension, there was no significant associations between each genotype of C9orf3 and the prevalence of hypertension. It is well-known that inhibitors of the renin-angiotensin system delay the progression of diabetic kidney disease^32,33^. Furthermore, numerous studies have observed activation of the RAS in subjects with type 2 diabetes^34–37^. RAS and type 2 diabetes are closely related. Thus, the significant association of the C allele was limited to subjects with type 2 diabetes. This may be due to the significant role that RAS has in the diabetic state.

There were several limitations to the present study. The sample number in this study was relatively small. Although population admixture is thought to contribute to concordant results among studies, our study only evaluated a Japanese population. Additionally, only one genetic variant of C9orf3 was evaluated.

In conclusion, the rs4385527 genetic variant of C9orf3 may significantly impact the onset of hypertension among subjects with type 2 diabetes. Based on the findings of our study, identifying a variant of C9orf3 in advance leads to improved predictive capacity for the development of hard endpoints such as cardiovascular and renal diseases. Therefore, a prospective study is required to examine the relationship between the C9orf3 genetic variant and the morbidity associated with hypertension.

### Ethics approval and consent to participate

The study was approved by the ethics committee of Fukui University (n. 13.1/14.2) and written informed consent was obtained prior to enrollment of all participants.

### Consent for publication

Not applicable.

## Data Availability

All data generated or analysed during this study are included in this published article.

## Abbreviations

RAS: Renin-angiotensin system
C9orf3: Chromosome 9 open reading frame 3
CYP11B2: Aldosterone synthase
PCOS: Polycystic ovarian syndrome
